# Myricetin Potentiates Antibiotics Against Resistant *Pseudomonas aeruginosa* by Disrupting Biofilm Formation and Inhibiting Motility Through FimX-Mediated c-di-GMP Signaling Interference

**DOI:** 10.3390/biology14070859

**Published:** 2025-07-15

**Authors:** Derong Zeng, Fangfang Jiao, Yuqi Yang, Shuai Dou, Jiahua Yu, Xiang Yu, Yongqiang Zhou, Juan Xue, Xue Li, Hongliang Duan, Yan Zhang, Jingjing Guo, Wude Yang

**Affiliations:** 1 College of Pharmacy, Guizhou University of Traditional Chinese Medicine, Guiyang 550025, China; yangwude476@gzy.edu.cn (W.Y.); 2 Centre in Artificial Intelligence Driven Drug Discovery, Faculty of Applied Sciences, Macao Polytechnic University, Macao, China; 3 School of Basic Medicine, Guizhou University of Traditional Chinese Medicine, Guiyang 550025, China; 4 The Second Affiliated Hospital of Guizhou University of Traditional Chinese Medicine, Guiyang 550025, China

**Keywords:** *Pseudomonas aeruginosa*, antibiotic resistance, biofilm, c-di-GMP, FimX, myricetin

## Abstract

*Pseudomonas aeruginosa* biofilm formation is critical to antibiotic resistance and persistence. Targeting cyclic di-GMP (c-di-GMP) signaling, a master biofilm formation and virulence regulator, presents a promising strategy to combat resistant bacterial infections. Myricetin, a natural polyphenolic flavonoid with documented antimicrobial and anti-biofilm activities, may enhance antibiotic efficacy against *Pseudomonas aeruginosa*. This study evaluated the synergistic effects of myricetin combined with azithromycin, ciprofloxacin, or cefdinir against both standard and drug-resistant *Pseudomonas aeruginosa* strains. Antibacterial activity, biofilm disruption, and motility inhibition were experimentally assessed, while molecular dynamic (MD) simulations elucidated myricetin’s molecular mechanism of action. Our results suggested that myricetin synergistically potentiated all three antibiotics, reducing c-di-GMP synthesis by 28% (azithromycin), 57% (ciprofloxacin), and 30% (cefdinir). It enhanced bactericidal effects, suppressed biofilm formation, and impaired swimming, swarming, and twitching motility. Computational analyses revealed that myricetin binds allosterically to FimX very well, a key regulator in the c-di-GMP signaling pathway. Hence, myricetin may act as a c-di-GMP inhibitor, reversing biofilm-mediated resistance in *Pseudomonas aeruginosa* and augmenting antibiotic efficacy. This integrated experimental and computational approach provides a framework for developing anti-virulence and antibiotic combination therapies against recalcitrant Gram-negative pathogens.

## Simple Summary

The emergence of resistant *Pseudomonas aeruginosa* has posed a significant challenge to global healthcare systems. Through systematic antibacterial and anti-biofilm experiments, we demonstrated that myricetin, a natural flavonoid compound derived from *Polygonum capitatum*, exhibited synergistic activity with conventional antibiotics against drug-resistant *Pseudomonas aeruginosa* clinical isolates. Molecular docking and molecular dynamics simulations further revealed that myricetin inhibited biofilm formation and decreased bacterial motility through specific targeting of the FimX-mediated c-di-GMP signaling network. This integrated approach combining experimental and computational methodologies establishes a robust foundation for developing natural compounds capable of potentiating antibiotic efficacy against resistant Gram-negative pathogens.

## 1. Introduction

The escalating resistance of *Pseudomonas aeruginosa* (*P. aeruginosa*) to the four principal antibiotic classes, such as aminoglycosides, quinolones, beta-lactams, and carbapenems, widely used in clinical settings, presents a significant global concern [1]. The World Health Organization (WHO) classifies this bacterium as one of the top three critical antibiotic-resistant pathogens, placing it on the high-priority list for the development of novel antibiotics [2,3,4]. Multidrug-resistant (MDR) strains of *P. aeruginosa* are associated with a broad spectrum of infections acquired both in community settings and healthcare facilities, including urinary tract infections, pneumonia, surgical site infections, bacteremia, endocarditis, burn infections, and complications related to COVID-19 [5,6]. According to reports in Healthcare Infectious Disease Reports, the global mortality rate for patients infected with *P. aeruginosa* stands at 20% but escalates to 30–50% for those suffering from ventilator-associated pneumonia and bacteremia [7]. The survival, colonization, multidrug resistance, and pathogenicity of *P. aeruginosa* are highly relevant to the formation of biofilms, which are intricate structures composed of attached or aggregated bacterial species and are implicated in approximately 65% of infectious diseases and over 80% of chronic infections [8,9]. Accordingly, developing alternative strategies to address biofilm-associated *P. aeruginosa* infections has become imperative.

Cyclic di-GMP (c-di-GMP) serves as a universal secondary messenger within bacterial cells, regulating a range of functions including biofilm formation, motility, adhesion, and antibiotic resistance [10,11]. For *P. aeruginosa*, biofilm development is intricately linked to cellular motility mechanisms such as swimming mediated by flagella, swarming regulated by both flagella and pili, and twitching motility mediated by type IV pili [12,13,14]. It is well established that low concentrations of c-di-GMP enhance cellular motility and hinder biofilm formation, whereas high concentrations of c-di-GMP promote the synthesis of adhesins and extracellular polysaccharides, facilitating biofilm formation and simultaneously inhibiting cellular motility [15,16,17]. FimX, a dual GGDEF-EAL domain protein, serves as a c-di-GMP receptor in *P. aeruginosa*, pivotal for regulating both twitching motility and biofilm formation [18,19,20]. At low c-di-GMP concentrations, FimX binds with high affinity to c-di-GMP via its EAL domain, triggering conformational shifts in the REC domain and adjacent N-terminal linkers. This action enhances the interaction with ATPase PilB and facilitates the assembly of type IV pili; however, at high c-di-GMP concentrations, FimX is not required [20,21,22]. Consequently, targeting the regulation of c-di-GMP to disrupt or inhibit biofilm formation and its associated molecular pathways contribute to enhancing antibiotic efficacy and effectively combating antibiotic resistance.

Previous studies have shown natural compounds, including triterpene saponins, coumarin, curcumin, cinnamic aldehyde, and sibiricose A5, can modulate c-di-GMP levels, thereby inhibiting bacterial biofilm formation [23,24,25,26]. In particular, cinnamic aldehyde and sibiricose A5 can synergize with antibiotics to modulate c-di-GMP concentrations. The traditional herb *Polygonum capitatum* has been conventionally utilized for treating urinary system disorders such as urinary tract infections and pyelonephritis. Pharmacological studies reveal its extract exhibits antimicrobial activity against multiple pathogens (*Escherichia coli*, *Staphylococcus aureus*, and *Helicobacter pylori*), along with demonstrated therapeutic potentials in inflammation modulation, cancer suppression, oxidative stress reduction, and blood glucose regulation [27,28]. Notably, its flavonoid-rich composition contains myricetin (MYR) as a key bioactive component that effectively inhibits calcium oxalate calculi formation, contributing to its nephroprotective effects [29].

Additionally, myricetin exhibits a broad spectrum of pharmacological properties, such as anti-inflammatory, analgesic, anti-tumor, antiviral, and antidiabetic properties [30,31,32]. Notably, it acts as a potent biofilm inhibitor, disrupting biofilm formation in multidrug-resistant *Staphylococcus aureus* without impeding bacterial growth, and diminishing *Helicobacter pylori*’s biofilm-forming capabilities. Furthermore, myricetin demonstrates synergistic interactions with antibiotics such as amoxicillin, clarithromycin, levofloxacin, metronidazole, and tetracycline, enhancing their efficacy against resistant pathogens [33]. However, its mechanism of action against *P. aeruginosa* remains underexplored, particularly its ability to interfere with c-di-GMP signaling, which is a central regulator of biofilm formation and virulence. Given the critical role of c-di-GMP in *P. aeruginosa* antibiotic resistance, targeting this pathway represents a promising strategy to restore antibiotic susceptibility.

**Figure 1:**
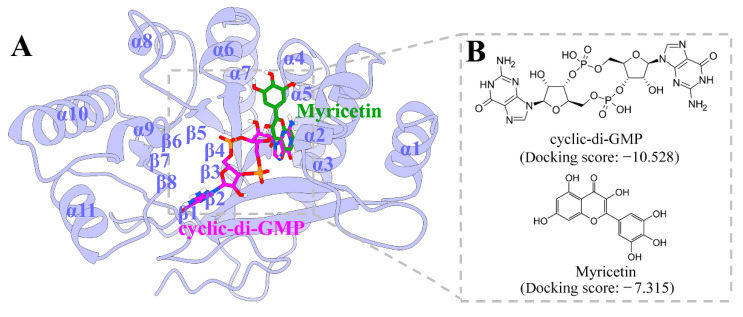
System information of FimX^EAL^ domain and its ligands. (**A**) Cartoon representation of the FimX^EAL^ domain, colored in purple. The c-di-GMP and myricetin molecules are shown as stick models, with carbon atoms in magenta for c-di-GMP and green for myricetin, oxygen atoms in red, nitrogen atoms in blue, and phosphorus atoms in orange. (**B**) Chemical structures and docking scores (in kcal/mol) of c-di-GMP and myricetin.

In this study, we investigated the synergistic effects of myricetin combined with azithromycin (AZM), ciprofloxacin (CIP), and cefdinir (CFD) against both wild-type and multidrug-resistant *P. aeruginosa* strains. Using a combination of experimental approaches, including broth microdilution, checkerboard assays, biofilm quantification, motility assays, and scanning electron microscopy, we evaluated myricetin’s ability to enhance antibiotic activity, disrupt biofilms, and impair bacterial motility. Complementing these findings, molecular docking (Figure 1) and dynamics simulations reveal that myricetin binds strongly to FimX, a key c-di-GMP effector, and thereby may interfere with c-di-GMP-mediated signaling. Our results demonstrate that myricetin significantly reduces c-di-GMP levels and potentiates the bactericidal effects of all the three antibiotics. These findings highlight myricetin’s potential as a c-di-GMP inhibitor capable of reversing biofilm-mediated resistance in *P. aeruginosa*. By integrating experimental and computational approaches, this study provides a framework for developing flavonoid-antibiotic combination therapies against multidrug-resistant Gram-negative pathogens, offering a novel strategy to address the global antibiotic resistance crisis.

## 2. Materials and Methods

### 2.1. Chemicals and Bacterial Strains

Myricetin and antibiotics (azithromycin, ciprofloxacin, and cefdinir) were purchased from Vicci Biotech Co., Ltd. (Chengdu, China). LB nutrient agar, broth, additional agar, crystal violet, phosphate buffered saline (PBS), and dimethyl sulfoxide (DMSO) were purchased from Beijing Solepol Science and Technology Co., Ltd. (Beijing, China) and Chuandong Chemical (Group) Co., Ltd. (Chongqing, China). MH culture medium was purchased from Hangzhou Microbial Reagent Co., Ltd. (Hangzhou, China), while tryptone and yeast powder were supplied by Oxoid. Additionally, glucose and sodium chloride were purchased from Tianjin Zhiyuan Chemical Reagent Co., Ltd. (Tianjin, China), and Tianjin Kemio Chemical Reagent Co., Ltd. (Tianjin, China), respectively.

*P. aeruginosa* ATCC 9027, *E. coli* CICC 10389, and *E. faecalis* ATCC 19,433 were obtained from the Biopharmaceutical Laboratory at the School of Pharmacy, Guizhou University of Traditional Chinese Medicine. Four clinical isolates of *P. aeruginosa*, seven clinical isolates of *E. coli*, and five clinical isolates of *E. faecalis* were provided by the Second Clinical Affiliated Hospital of Guizhou University of Traditional Chinese Medicine. All strains were cultured on LB agar plates at 37 °C for 24 h. A well-grown single colony was then transferred to 100 mL of LB broth and incubated at 37 °C with shaking at 220 rpm for 16 h to achieve a bacterial suspension with an optical density (OD) at 600 nm of 0.1, which was subsequently diluted 1:100 in LB broth for further experiments.

### 2.2. In Vitro Antibacterial Assay

#### 2.2.1. Minimum Inhibitory Concentration (MIC)

The MICs of myricetin and various antibiotics were determined against each bacterium using the broth microdilution method, adhering to the American Clinical and Laboratory Standards Institute (CLSI) guidelines [34]. In brief, test compounds were serially diluted two-fold (1024 μg/mL to 2 μg/mL) in 96-well plates preloaded with 100 μL LB broth per well, followed by addition of 100 μL bacterial suspension at 10^6^ CFU/mL. The plates were incubated statically at 37 °C for 24 h and MIC was identified by observing the lowest drug concentration that prevented visible bacterial growth, indicated by a lack of turbidity. Each experiment was conducted in triplicate for accuracy.

#### 2.2.2. Collaborative Research

The synergistic effects of antibiotics combinations with myricetin on *P. aeruginosa* ATCC 9027 were qualitatively assessed using the Kirby–Bauer disk diffusion method [25]. The blank drug-sensitive paper (6 mm × 1 mm) was immersed in solutions of myricetin (200 μg/mL), azithromycin (15 μg/mL), ciprofloxacin (5 μg/mL), cefdinir (30 μg/mL), and combinations of myricetin with each antibiotic. The diluted bacterial suspension was uniformly spread on LB agar plates. Following drying, the drug-containing disks were placed equidistantly on the plates. The plates were then incubated at 37 °C for 18–24 h.

The synergistic effect of myricetin and antibiotics, as well as the fractional inhibitory concentration index (FICI), was evaluated using the checkerboard microdilution method [35]. Serial two-fold dilutions of myricetin and antibiotics were performed in the horizontal and vertical directions, respectively. Then, 100 μL of *P. aeruginosa* suspension (10^6^ CFU/mL) was added to each well containing varying concentrations of the reagents and the plates were incubated at 37 °C for 18 h. The FICI for each antibiotic in combination with myricetin was calculated using the formula below:

$$\mathrm{FICI}={\;\mathrm{FICI}}_{A}+{\mathrm{FICI}}_{B}={\;\mathrm{MIC}}_{\mathrm{AB}}/{\mathrm{MIC}}_{A}+{\mathrm{MIC}}_{\mathrm{BA}}/{\mathrm{MIC}}_{B}$$

In this context, ‘A’ represents myricetin, while ‘B’ denotes antibiotics. The FICI is classified as follows: FICI ≤ 0.5 indicates a synergistic effect; 0.5 < FICI < 1 denotes partial synergy; FICI = 1 suggests an additive interaction; FICI > 1 indicates antagonism.

#### 2.2.3. Time-Kill Curves

To further investigate the synergistic antibacterial activity of myricetin with various antibiotics, we assessed the time-kill curves of the combination of myricetin and each antibiotic during the exponential growth phase of *P. aeruginosa* strain ATCC 9027 and clinical isolate PA01 [36]. LB bacterial cultures, adjusted to a concentration of 10^6^ CFU/mL, were treated with myricetin and each antibiotic either singly or in combination (1/4 MIC), followed by incubation at 37 °C. Subsequently, at predetermined time points (0, 6, 12, and 24 h), each sample was serially diluted in PBS, plated on MHA agar, and incubated for 24 h at 37 °C. All experiments were conducted in triplicate.

### 2.3. Determination of Anti-Biofilm Activity of Myricetin Combined with Antibiotics Against P. aeruginosa

#### 2.3.1. Quantitative Detection of Resistance to Biofilm Formation

Following the literature with minor modifications, we assessed the inhibitory effects of myricetin combined with antibiotics on bacterial biofilms using crystal violet staining [37]. Specifically, the biofilm formation capabilities of *P. aeruginosa* ATCC 9027 and clinical isolate PA01 were examined. A 100 μL bacterial suspension (10^6^ CFU/mL) was inoculated into each well of a 96-well plate, which was treated with 100 μL of drug solutions at different concentrations (MIC, 1/2 MIC) and incubated at 37 °C for 36 h. Post-incubation, planktonic cells were carefully removed and biofilms were washed thrice with PBS to eliminate non-adherent bacteria, followed by fixation with methanol for 15 min. Afterwards, the excess stain was rinsed off with PBS. Once dried, the biofilms were solubilized with 33% acetic acid for 15 min and the optical density (OD) at 590 nm was measured using a microplate reader. Results are expressed as mean values ± SD from at least three independent biological experiments.

#### 2.3.2. Morphological Analysis of Biofilm Development via Light Microscopy

The qualitative effects of myricetin combined with various antibiotics on biofilm formation were assessed using light microscopy [38]. *P. aeruginosa* ATCC 9027 and clinical isolate PA01 biofilms were cultivated on glass slides in 24-well plates with a 1/4 MIC of myricetin and antibiotics for 24 h. The biofilms were then stained with 0.4% crystal violet for 1 min, rinsed to remove excess dye and impurities, and air-dried. Observations were made using an Olympus CX41 microscope and images were captured with an attached digital camera.

#### 2.3.3. Biofilm Adherence Evaluated by Scanning Electron Microscopy (SEM)

SEM was utilized to assess the biofilm adhesion of *P. aeruginosa* ATCC 9027 following combined treatment with myricetin and various antibiotics. Sterile slides were placed in a 24-well plate containing a 10^6^ CFU/mL bacterial suspension, supplemented with 1/4 MIC of myricetin and antibiotics, and incubated at 37 °C for 48 h. After supernatant removal, the slides were gently rinsed thrice with sterile water and fixed overnight in 2.5% (*w*/*v*) glutaraldehyde solution. Subsequently, the glutaraldehyde was washed off the slide surface three times with ultrapure water (15 min per wash). The slides underwent dehydration through a graded series of ethanol concentrations (30%, 50%, 70%, and 90%, each for 15 min) followed by three 15-min washes in 100% ethanol. Lastly, the slides were gold-sputtered using a JEOL Smart Coater and examined under a JEOL JSM-IT700HR SEM.

### 2.4. Assessing the Impact of Myricetin Combined with Antibiotics on the Motility of P. aeruginosa

Bacterial swimming, swarming, and twitching motility were measured on three media in the presence or absence of subinhibitory concentrations of myricetin (1/8 MIC) and antibiotics (1/4 MIC) as previously described [39,40]. Add 2 uL of bacterial culture of *P. aeruginosa* (ATCC 9027 and PA01) to swimming agar plates (1.0% (*w*/*v*) tryptone, 0.5% (*w*/*v*) sodium chloride, and 0.3% (*w*/*v*) agar), and swarming agar plates (1% (*w*/*v*) glucose, 0.5% (*w*/*v*) peptone, 0.2% (*w*/*v*) yeast extract, 0.5% (*w*/*v*) agar). Incubate the plates at 37 °C for 24 h and measure the diameter of the motor zone. Use a sterile toothpick to inoculate single colonies onto twitching agar plates (1.0% (*w*/*v*) tryptone, 0.5% (*w*/*v*) yeast extract, 1.0% (*w*/*v*) sodium chloride and 1.0% (*w*/*v*) agar) at the bottom of the center and incubate the plate upside down at 37 °C for 24 h. Then, the agar was carefully removed with a toothpick and the diameter was determined by staining with 1% crystal violet for 20 min and then the excess stain was washed away.

### 2.5. Myricetin Combined with Antibiotics for the Extraction and Quantification of Intracellular c-di-GMP in P. aeruginosa

Intracellular c-di-GMP levels were quantified according to previously established methodologies [41]. *P. aeruginosa* ATCC 9027 cultured to the exponential growth phase was inoculated into LB medium supplemented with myricetin at a concentration of 1/8 MIC and antibiotics, followed by shaking incubation at 37 °C until the OD_600_ reached 0.6. Aliquots (2 mL) were centrifuged at 10,000× *g* for 5 min at 4 °C, with resultant cell pellets resuspended in ice-cold PBS. Subsequent thermal lysis was achieved through 5-min incubation at 100 °C, followed by ultrasonication in an ice-water bath (100% power, 37 kHz) for 15 min. After centrifugation, c-di-GMP-containing supernatants were collected for analysis using a commercial ELISA kit (Mskbio, Beijing, China). Total protein concentrations were determined using a Pierce bicinchoninic acid (BCA) protein assay kit (Solarbio, Beijing, China), with final c-di-GMP levels normalized to pmol/mg protein.

### 2.6. Statistical Analysis

All experimental trials were conducted at least three times. Results were reported as the mean ± standard deviation (SD) and a one-way ANOVA was performed using SPSS Statistics version 27. *p* ≤ 0.05 was deemed statistically significant.

## 3. Results

### 3.1. Antibacterial Activity of Myricetin and Antibiotics

In this study, we applied the broth microdilution method to determine the minimum inhibitory concentrations of myricetin and three antibiotics (azithromycin, ciprofloxacin, and cefdinir) against various common clinical bacterial strains (Table 1). As can be seen, myricetin exhibited moderate broad-spectrum antibacterial effects, with MICs ranging from 256 μg/mL to 1024 μg/mL. These findings demonstrated myricetin’s antimicrobial properties against diverse microorganisms, aligning with previous research [31]. In contrast, azithromycin and ciprofloxacin displayed more potent inhibitory actions, particularly ciprofloxacin, which showed MICs between 0.5 µg/mL and 64 µg/mL against most strains, significantly outperforming myricetin and indicating robust broad-spectrum antibacterial capabilities. Cefdinir’s bacteriostatic effect was somewhat less potent but still markedly superior to that of natural compounds. Among the bacteria tested, *P. aeruginosa* showed the highest resistance, likely due to its ability to produce various types of exopolysaccharides (EPSs) and related biofilm structures that enhance its antibiotic resistance and aid in evading host immune responses.

### 3.2. Synergistic Activity of Myricetin Combined with Antibiotics Against P. aeruginosa

For *P. aeruginosa* ATCC 9027, myricetin demonstrated synergistic activity when combined with each of the three antibiotics tested, as presented in Table 2 and Figure S1. The corresponding enhancements in inhibition zone diameters were 7.7 mm, 4.4 mm, and 2.1 mm.

To further assess the synergistic effects of myricetin with antibiotics, we examined their combined antibacterial actions against *P. aeruginosa* and four resistant clinical strains via the checkerboard microdilution technique. As shown in Table 3, the myricetin-azithromycin-cefdinir combination exhibited significant synergy (FICI = 0.125–0.75), reducing myricetin’s MIC value by 2- to 16-fold. Similarly, the MIC values of azithromycin and cefdinir decreased significantly, ranging from 8- to 32-fold and 4- to 32-fold, respectively. Moreover, while myricetin and ciprofloxacin showed no synergistic effect on the clinical strains PA02 and PA03 of *P. aeruginosa*, they were effective against the standard strains ATCC 9027 and clinical strains PA01 and PA04 (FICI = 0.28125–1), with MIC values decreased by 2- to 16-fold. These findings suggest a cooperative antibacterial activity of myricetin and antibiotics against *P. aeruginosa* and its clinical strains, indicating potential interactions that could enhance antibiotic efficacy against these pathogens.

**Figure 2:**
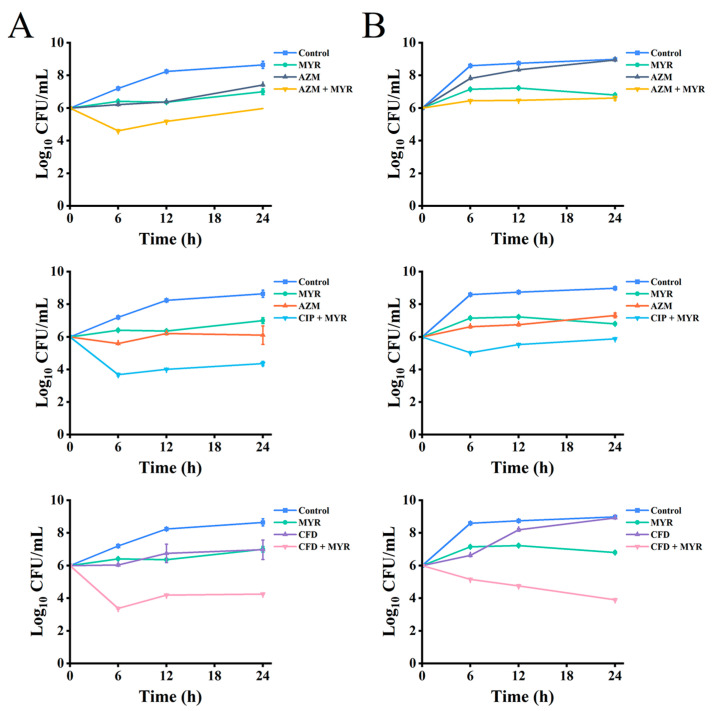
Time-kill curves of myricetin and antibiotics at 1/4 MIC, tested individually and in combination against *P. aeruginosa* ATCC 9027 (**A**) and PA01 (**B**). Data shown are means ± SD from a minimum of three independent experiments.

To further explore the synergistic antibacterial potential of myricetin, we used *P. aeruginosa* ATCC 9027 and the clinically resistant strain PA01 as model organisms. We assessed the effectiveness of myricetin combined with azithromycin, ciprofloxacin, and cefdinir by analyzing time-kill curves and pharmacokinetic properties (Figure 2). The results indicated that at sub-MIC levels (1/4 MIC), myricetin and the antibiotics individually had limited effects on both the standard and the drug-resistant strains, with no significant reduction in colony-forming units (Log_10_ CFU/mL) over time. However, the combined treatment with myricetin and the antibiotics significantly outperformed the individual treatments, exhibiting synergistic effects at various time points.

### 3.3. Synergistic Anti-Biofilm Activity of Myricetin Combined with Antibiotics Against P. aeruginosa

**Figure 3:**
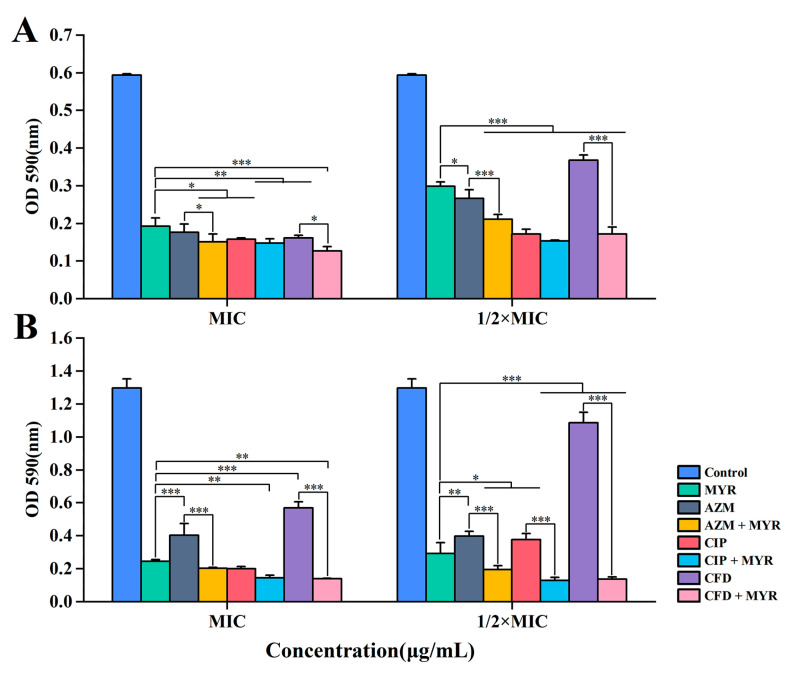
The OD values at 590 nm of varying concentrations of myricetin and antibiotics were measured against *P. aeruginosa* ATCC 9027 (**A**) and PA01 (**B**) after 36 h. Data are presented as the mean ± standard deviation (SD) from at least three independent experiments (*, *p* < 0.05; **, *p* < 0.01; ***, *p* < 0.001).

The aforementioned experimental findings demonstrate that myricetin exhibits notable antibacterial activity and can synergistically enhance the inhibitory effects of azithromycin, ciprofloxacin, and cefdinir. To further investigate its potential synergistic anti-biofilm efficacy against *P. aeruginosa*, a crystal violet staining assay was conducted. As illustrated in Figure 3, after 36 h of treatment, myricetin at varying concentrations significantly attenuated biofilm formation in both the standard strain *P. aeruginosa* ATCC 9027 (Figure 3A) and the clinically resistant strain PA01 (Figure 3B). Notably, synergistic interactions with antibiotics were observed, leading to a marked reduction in biofilm-forming capacity. Among the tested antibiotic combinations, myricetin exhibited the most pronounced synergistic anti-biofilm activity with cefdinir, while its synergy with ciprofloxacin was comparatively less marked. Intriguingly, the synergistic inhibition of biofilm formation was more potent in the clinically resistant strain PA01 than in the standard strain, particularly at sub-MIC concentrations (1/2 MIC). Furthermore, myricetin alone demonstrated superior anti-biofilm activity against the resistant strain PA01 compared to the individual application of the three antibiotics. These results underscore the therapeutic potential of myricetin as an adjuvant agent to potentiate conventional antibiotics, especially in combating biofilm-associated infections caused by drug-resistant pathogens.

### 3.4. Microscopic in Situ Visualization of Biofilm Formation by P. aeruginosa

**Figure 4:**
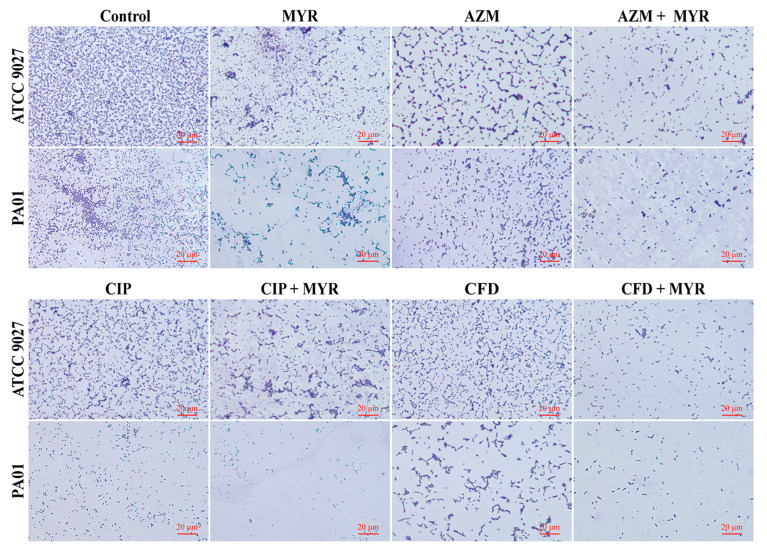
The optical microscope images of the crystal violet staining of biofilms in *P. aeruginosa* ATCC 9027 and PA01 after combination treatment with myricetin and antibiotics at 1/4 MIC concentration.

Optical microscope and SEM were utilized to assess the efficacy of myricetin in combination with antibiotics in inhibiting the biofilms of *P. aeruginosa*. Figure 4 illustrates that treatment of the standard *P. aeruginosa* ATCC 9027 with either myricetin at 1/4 MIC concentration or individual antibiotics (azithromycin, ciprofloxacin, or cefdinir) alone resulted in reduced biofilm adhesion. Concurrent administration of azithromycin or cefdinir with myricetin significantly reduced the biofilm attachment to glass slides. In contrast, ciprofloxacin combined with myricetin did not show notable biofilm reduction in this strain. For the drug-resistant strain PA01, a similar reduction in biofilm adhesion was observed, aligning with results from crystal violet staining. The experiments confirm a synergistic effect of myricetin with azithromycin and cefdinir against biofilms of the standard strain.

**Figure 5:**
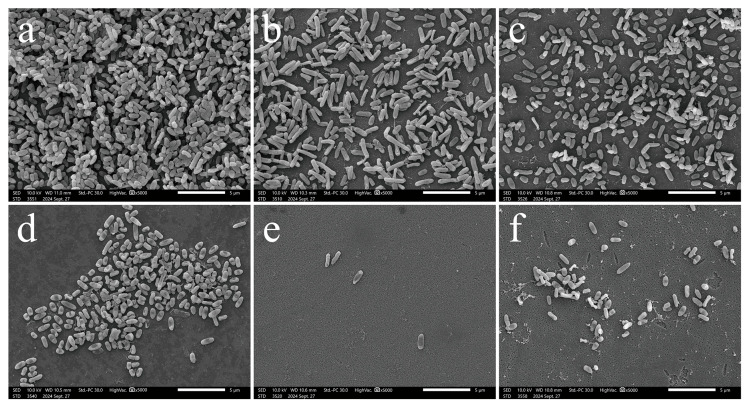
SEM images depict the inhibitory effects on biofilm formation of *P. aeruginosa* ATCC 9027 by myricetin, azithromycin, and cefdinir at 1/4 MIC concentration. (**a**) Control; (**b**) 1/4 MIC azithromycin; (**c**) 1/4 MIC cefdinir; (**d**) 1/4 MIC myricetin; (**e**) 1/4 MIC (azithromycin + myricetin); (**f**) 1/4 MIC (ceftinir + myricetin).

SEM confirmed that myricetin enhances the anti-biofilm properties of these antibiotics (Figure 5). Untreated cells of *P. aeruginosa* displayed typical morphology and initiated biofilm formation. Treatment with azithromycin or cefdinir alone led to sparser colonies with altered cell shapes; azithromycin treatment elongated cells, as seen under a light microscope. Myricetin treatment, both alone and in combination, significantly inhibited biofilm adhesion, dispersed cells, and induced surface collapse, shrinkage, and lysis. These effects demonstrate its dual antibacterial and anti-biofilm activity, likely mediated by cellular surface damage. Myricetin was also shown to augment the effectiveness of azithromycin and cefdinir in reducing biofilm formation by *P. aeruginosa*.

### 3.5. The Combination of Myricetin and Antibiotics Effectively Restricts the Motility of P. aeruginosa

**Figure 6:**
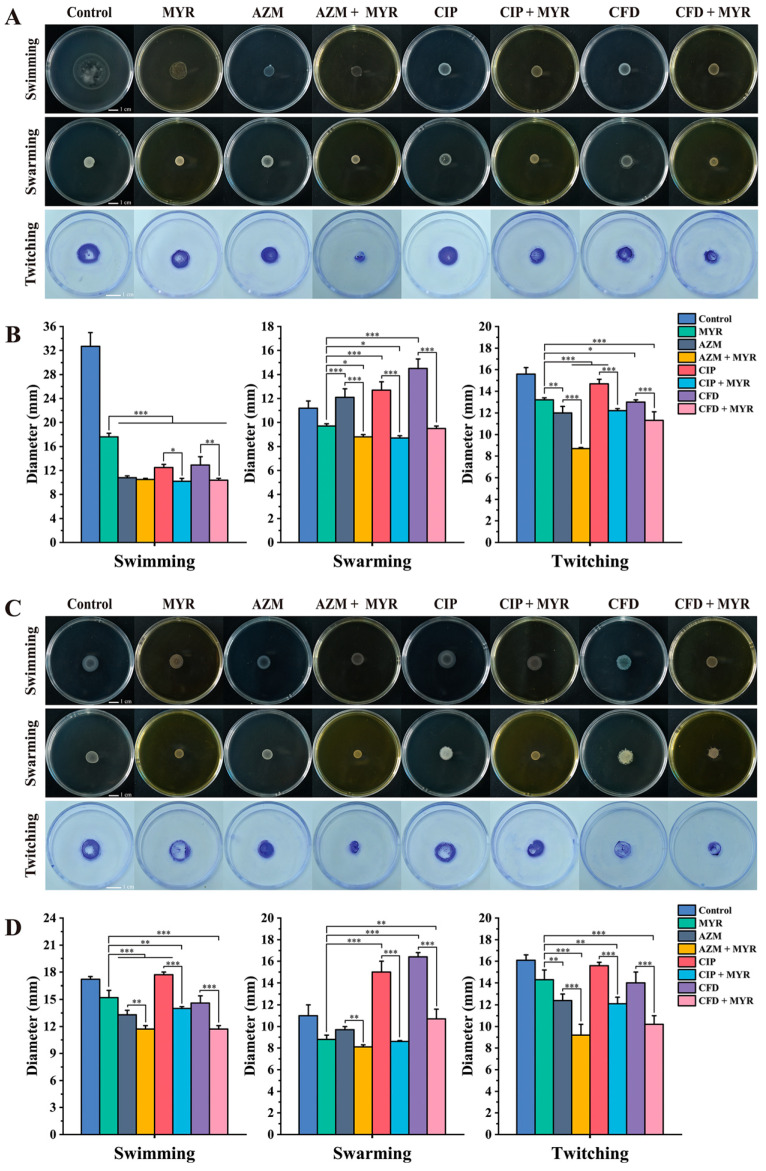
Effects of myricetin at 1/8 MIC concentration and antibiotics at 1/4 MIC concentration on the motility of *P. aeruginosa* ATCC 9027 and PA01. Swimming, swarming, and twitching motility of (**A**) ATCC 9027 group and (**C**) PA01 group. Statistics of swimming, swarming, and twitching motility diameters of (**B**) ATCC 9027 group and (**D**) PA01 group. Data are expressed as mean ± SD of at least three independent experiments (*, *p* < 0.05; **, *p* < 0.01; ***, *p* < 0.001).

Bacterial motility plays a crucial role in biofilm adhesion and development. To further investigate biofilm formation, we tested the effects of sub-MIC concentrations of myricetin (1/8 MIC) combined with antibiotics (1/4 MIC) on the motility (swimming, swarming, and twitching) of *P. aeruginosa* ATCC 9027 and the clinical multidrug-resistant strain PA01. As shown in Figure 6, myricetin was found to reduce the motility of *P. aeruginosa* and significantly enhance the inhibitory effects of antibiotics. The most notable synergistic inhibition was observed in swarming motility, followed by twitching. In *P. aeruginosa* ATCC 9027, the synergistic inhibition rates of myricetin combined with azithromycin on swimming, swarming, and twitching motility were 3.09%, 27.35%, and 27.70%, respectively; with ciprofloxacin, 18.62%, 31.23%, and 16.97%, respectively; and with cefdinir, 19.33%, 34.40%, and 13.30%. For the resistant strain PA01, the synergistic inhibition rates of myricetin combined with azithromycin on swimming, swarming, and twitching motility were 11.78%, 16.44%, and 25.81%, respectively; with ciprofloxacin, 21.24%, 42.54%, and 22.06%, respectively; and with cefdinir, 19.86%, 34.96%, and 26.97%, respectively.

### 3.6. Myricetin Synergistically Inhibits the Synthesis of Intracellular c-di-GMP in Combination with Antibiotics

**Figure 7:**
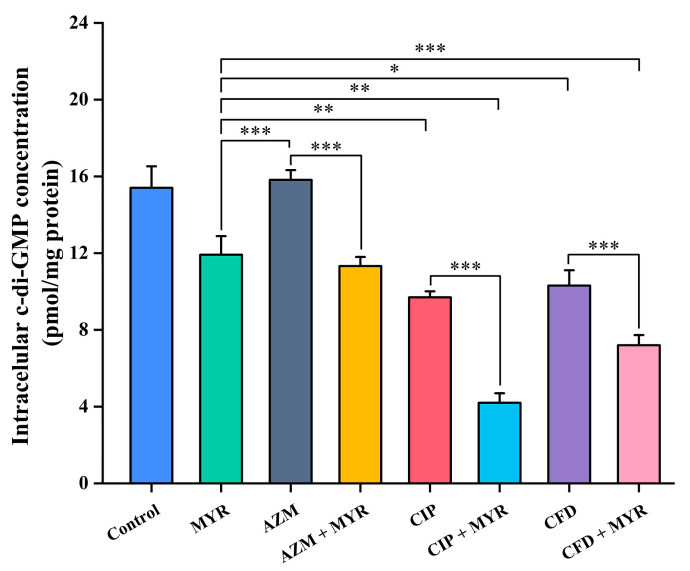
Intracellular c-di-GMP levels in *P. aeruginosa* ATCC 9027 after treatment with myricetin and antibiotics at 1/8 MIC concentration. Data are expressed as the mean ± SD of at least three independent experiments (*, *p* < 0.05; **, *p* < 0.01; ***, *p* < 0.001).

The c-di-GMP, as a core signaling molecule in bacteria, is widely involved in regulating various physiological processes such as the cell cycle, differentiation, biofilm dynamics, motility, antibiotic resistance, and host colonization. Therefore, this study employed the ELISA method to measure the impact of myricetin combined with different antibiotics at a concentration of 1/8 MIC on the intracellular c-di-GMP levels in *P. aeruginosa* ATCC 9027. As shown in Figure 7, myricetin inhibits the production of c-di-GMP in *P. aeruginosa* with an inhibition rate of 23% and it synergistically enhances the inhibitory effect of antibiotics on c-di-GMP synthesis (*p* < 0.001). Compared to antibiotics used alone, myricetin showed synergistic inhibition rates of 28%, 57%, and 30% for azithromycin, ciprofloxacin, and cefdinir, respectively.

### 3.7. The Potential Mechanism of Action of Myricetin Targeting FimX^EAL^ via c-di-GMP Signaling Disruption

#### 3.7.1. Significant Reduction in FimX^EAL^ Flexibility due to Ligand Binding

Complementing our experimental findings, computational approaches have been employed to investigate the potential mechanism of action of myricetin targeting FimX^EAL^, a key c-di-GMP effector, via c-di-GMP signaling disruption. While *P. aeruginosa* contains multiple c-di-GMP effectors, such as FleQ and BrlR, our molecular docking analyses revealed that myricetin exhibits preferential binding affinity for FimX (Figure S2 and Figure 1B). This selectivity is particularly important as it suggests that myricetin could serve as a specific modulator of FimX-mediated signaling pathways without broadly disrupting all c-di-GMP-dependent processes in the cell. To further explore the underlying mechanism, molecular dynamics simulations were performed. To assess the convergence of MD trajectories for the three FimX^EAL^ systems (FimX^EAL^-apo, FimX^EAL^-CDG, and FimX^EAL^-MYR), the root-mean-square deviation (RMSD) of the Cα atoms of FimX^EAL^ was first calculated relative to their initial structures for each system. As illustrated in Figure S3, the RMSD values for all simulations remained below 2 Å during the final 100 ns, indicating a high degree of stability for the FimX^EAL^ systems throughout the simulations. Consequently, the average trajectories of the last 100 ns from three independent simulations were selected for further analysis.

**Figure 8:**
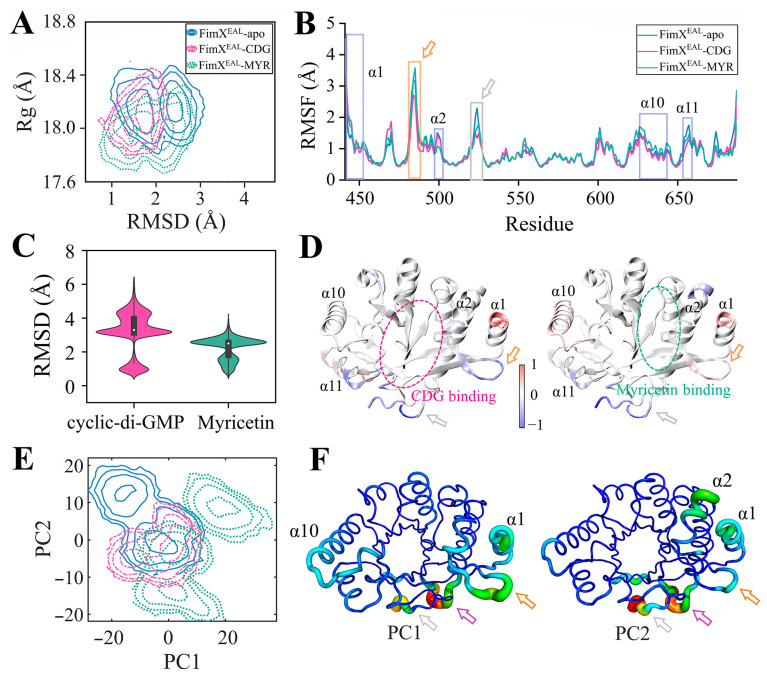
Structural and functional analysis of the FimX^EAL^ domain and its interactions with c-di-GMP and myricetin. (**A**) Comparison of the 2D potential of mean force (PMF) profiles constructed based on the RMSD and Rg of the FimX^EAL^ domain in its apo, c-di-GMP-bound, and myricetin-bound states during MD simulations. (**B**) RMSF values of the FimX^EAL^ domain residues in the apo, c-di-GMP-bound, and myricetin-bound states. Key regions with significant fluctuations are labeled as α1, α2, α10, α11, and two flexible loops indicated by the orange and gray arrows. (**C**) Comparison of the RMSD values of c-di-GMP and myricetin molecules during MD simulations. (**D**) The RMSF differences are exhibited on the FimX^EAL^ framework based on a color scale (high flexibility, red; low flexibility, blue): FimX^EAL^-c-di-GMP and FimX^EAL^-apo systems (left); FimX^EAL^-MYR and FimX^EAL^-apo systems. All results were obtained considering three replicates. (**E**) Free energy profiles according to PC1 and PC2. (**F**) Sausage representations of PC1 and PC2, where the width and color (ranging from blue to red) illustrate the conformational differences. The three arrows indicate key functional regions of the protein: the orange arrow indicates the α0 helix, the grey arrow points to C-terminal linker region, and the purple arrow highlights the α3 helix. Detailed motions are provided in Supplementary Movies S1 (PC1) and S2 (PC2).

To further elucidate the impact of ligand binding on the dynamic behavior of the FimX^EAL^ protein, the two-dimensional potential of mean force (PMF) profile was calculated based on RMSD and radius of gyration (Rg). As depicted in Figure 8A, the FimX^EAL^-apo system exhibited relatively higher RMSD and Rg values compared to the FimX^EAL^-CDG and FimX^EAL^-MYR systems, suggesting that the apo form of FimX^EAL^ adopts a more extended and less stable protein conformation. This instability may be attributed to the absence of ligand binding and stabilization. In contrast, the FimX^EAL^-CDG and FimX^EAL^-MYR displayed lower RMSD and Rg values with more concentrated distributions, indicating that the binding of c-di-GMP and myricetin ligands enhances the conformational stability of FimX^EAL^, maintaining it in a more compact state. By comparing the PMF distributions of FimX^EAL^-CDG and FimX^EAL^-MYR, we observed that the RMSD distribution for FimX^EAL^-MYR was slightly higher than that for FimX^EAL^-CDG, although their overall distribution patterns were similar. This phenomenon may be attributed to the comprehensive conformational sampling achieved in this study by considering three independent simulation trajectories. Notably, the Rg values of FimX^EAL^-MYR were significantly lower than those of FimX^EAL^-CDG, suggesting that myricetin may be more effective than c-di-GMP in stabilizing the overall conformation of FimX^EAL^, maintaining it in a more compact structural state.

Next, to investigate the impact of ligand binding on the conformational flexibility of FimX^EAL^, we calculated the root mean square fluctuations (RMSFs) of the Cα atoms for the three FimX^EAL^ systems using the last 100-ns MD trajectories. As shown in Figure 8B, the average RMSF profiles exhibited similar patterns across the three systems, with most regions displaying RMSF values below 1.0 Å, indicating the overall stability of the simulated systems. However, notable differences were observed in specific regions upon ligand binding.

In detail, compared to the apo state, the α1 helix exhibited significantly higher RMSF values in the presence of both c-di-GMP and myricetin. This suggests that ligand binding induces increased flexibility in this region, which may be essential for accommodating the ligands and facilitating conformational changes. In contrast, the binding site regions, including the α2 and α11 helices, as well as the two loops indicated by the orange and gray arrows, displayed reduced RMSF values in the ligand-bound systems compared to the apo state. These observations highlight the stabilizing effect of c-di-GMP and myricetin on the binding pocket of FimX^EAL^. To visualize the spatial distribution of the RMSF differences between the ligand-bound and apo systems, we mapped the RMSF changes onto the FimX^EAL^ protein structure (Figure 8D). The results demonstrate the increased dynamics in the α1 helix and decreased flexibility in the binding site upon ligand binding. The reduced conformational flexibility of the binding pocket suggests that the interactions between FimX^EAL^ and the ligands (c-di-GMP and myricetin) effectively stabilize this region, which may be crucial for maintaining the bound state and modulating protein function.

In addition, the RMSD values of the heavy atoms for both c-di-GMP and myricetin were monitored to explore the stability of ligand binding in the FimX^EAL^-CDG and FimX^EAL^-MYR systems over the last 100-ns MD trajectories (Figure 8C). Remarkably, myricetin exhibited significantly lower RMSD distributions in comparison to c-di-GMP, indicating a highly stable binding mode within the FimX^EAL^ binding site. This indicates a strong and persistent association between myricetin and the FimX^EAL^ protein, which could contribute to its effective modulation of FimX^EAL^ function.

To further investigate the conformational dynamics of the three FimX^EAL^ systems, PCA was performed by integrating the last 100-ns MD trajectories (Figure 8E,F). The free energy surfaces were constructed using the first two principal components (PC1 and PC2), which capture the dominant conformational changes in the systems. As depicted in Figure 8E, the conformational differences between the ligand-bound and apo systems are primarily concentrated along PC1 and PC2. Specifically, compared to FimX^EAL^-apo, the conformational motions of FimX^EAL^-CDG and FimX^EAL^-MYR are directed towards the positive direction of PC1. This corresponds to the contraction of α1, α2, and the three functional loops indicated by the arrows towards the protein’s internal center, resulting in a more compact and tighter binding pocket in FimX^EAL^ (Figure 8F and Movie S1). The contraction of these structural elements upon ligand binding suggests that the presence of c-di-GMP and myricetin induces a conformational rearrangement that favors a closed and stable binding pocket configuration.

In contrast, the conformational motions along PC2 primarily correspond to the outward extension of α1, α2, and the three functional loops. This implies that the apo state, which is positioned closer to the positive direction of PC2, exhibits a more open and extended conformation of the binding pocket (Figure 8F and Movie S2). The outward extension of these structural elements in the absence of ligands indicates a higher degree of flexibility and accessibility of the binding pocket in the apo state.

#### 3.7.2. The Binding Mechanism of FimX^EAL^ and Two Molecules (c-di-GMP and Myricetin)

Next, to quantitatively assess the binding affinity between the FimX^EAL^ protein and two ligands (c-di-GMP and myricetin), we performed MM-GBSA [42] calculations on the last 100-ns MD trajectories. The average binding free energies (Δ*G*_MM/GBSA_) and the individual contributions of each energy component are summarized in Table 4. As can be seen, the Δ*G*_MM/GBSA_ values were found to be −39.40 kcal/mol for the FimX^EAL^-CDG complex and −28.24 kcal/mol for the FimX^EAL^-MYR complex, indicating a strong binding affinity between FimX^EAL^ and both ligands.

Further decomposition of the binding free energy revealed that van der Waals (ΔE_vdw_) interactions serve as the primary driving force for the binding of c-di-GMP and myricetin to the FimX^EAL^ protein. The highly negative values of ΔE_vdw_ highlight the significant contributions of these energy components to the overall binding affinity. The nonpolar component of the solvation-free energy (ΔG_npol,sol_), which represents the hydrophobic effect and the entropy change associated with the burial of solvent-accessible surface area, was found to be slightly favorable for binding. The negative values of ΔG_npol,sol_ suggest that the hydrophobic interactions between the ligands and the protein contribute favorably to the binding affinity, albeit to a lesser extent compared to the electrostatic interaction.

**Figure 9:**
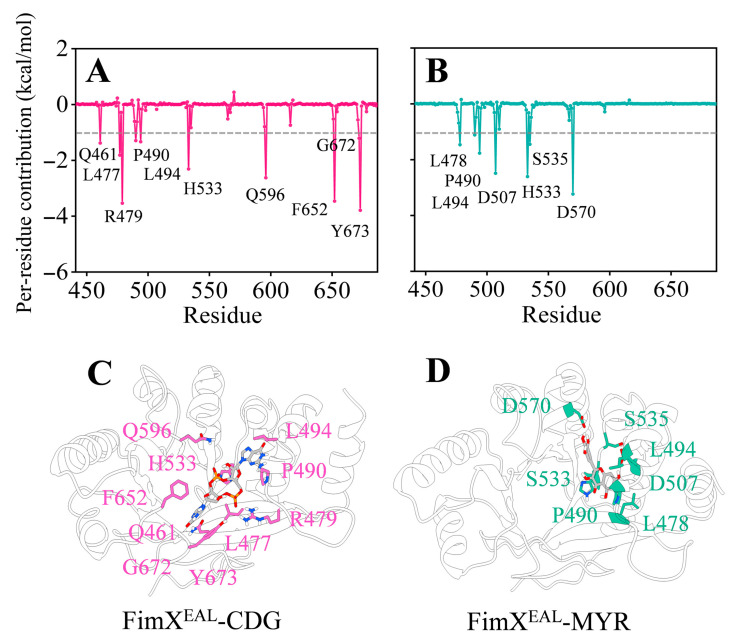
Per-residue contribution to the binding between FimX^EAL^ and the two molecules: (**A**) c-di-GMP; (**B**) myricetin. The binding modes between FimX^EAL^ and c-di-GMP (**C**) or myricetin (**D**). FimX^EAL^ and c-di-GMP are shown as grey cartoons and sticks, respectively. Key residues for the binding of c-di-GMP and myricetin are shown as sticks in pink and green, respectively.

Moreover, we performed a per-residue decomposition of the binding free energies to further explore the key residues contributing to the binding of c-di-GMP and myricetin to the FimX^EAL^ protein. As illustrated in Figure 9, hotspot residues (contributing less than −1 kcal/mol) were labelled in the FimX^EAL^-CDG and FimX^EAL^-MYR systems. Notably, residues P490, L494, and H533 were found to be common hotspot residues in both systems, suggesting their crucial role in ligand recognition and binding.

Interestingly, residues D507 and D570 exhibited exceptionally strong contributions (greater than −2 kcal/mol) in the FimX^EAL^-MYR system, while their contributions were not as significant in the FimX^EAL^-CDG system. As presented in Figure 9, it is evident that c-di-GMP exhibits stronger key residue contributions compared to myricetin. This difference can be attributed to the larger molecular scaffold of c-di-GMP, which enables it to interact with a greater number of protein residues within the binding pocket of FimX^EAL^. However, upon closer inspection of the binding site, it becomes apparent that the myricetin molecule occupies only a fraction of the binding cavity that accommodates c-di-GMP in the FimX^EAL^ protein. This observation raises an intriguing possibility: given the relatively spacious nature of the FimX^EAL^ binding pocket, it is plausible that two or more myricetin molecules could simultaneously interact with FimX^EAL^ during the actual binding process, potentially triggering the functional activation of the protein.

To further elucidate the molecular basis of ligand recognition and binding in the FimX^EAL^ system, we mapped the identified key residues onto the FimX^EAL^ protein. As depicted in Figure 9A,B, specific polar contacts between the ligands (c-di-GMP and myricetin) and the FimX^EAL^ protein were observed, providing insights into the structural determinants of ligand-protein interactions. In the FimX^EAL^-CDG complex (Figure 9C), polar contacts were established between c-di-GMP and several key residues, including Q461, L477, R479, P490, L494, H533, Q596, F652, G672, and Y673. These polar contacts contribute to the specific recognition and tight binding of c-di-GMP within the FimX^EAL^ binding pocket. Similarly, in the FimX^EAL^-MYR complex (Figure 9D), myricetin engages in polar contacts with a distinct set of key residues, including L478, P490, L494, D507, H533, S535, and D570. The presence of polar contacts between myricetin and these residues underscores their role in stabilizing the ligand within the binding pocket.

#### 3.7.3. The Ligand Binding Enhances the Internal Coupling of the FimX^EAL^ Protein

**Figure 10:**
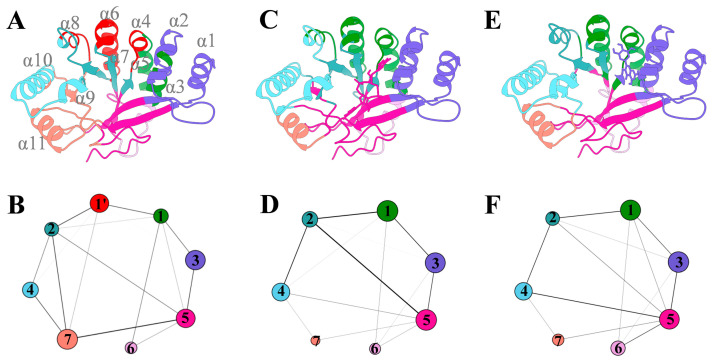
Dynamic community analysis for the three FimX^EAL^ systems: (**A**,**B**) FimX^EAL^-apo; (**C**,**D**) FimX^EAL^-CDG; (**E**,**F**) FimX^EAL^-MYR. Communities are visualized as cartoon representations (upper row) or circular diagrams (lower row) with consistent color schemes. The numbered nodes (1–7 and 1') in the circular diagrams correspond to distinct structural communities: 1 (α3 and α5 region), 2 (β4-β6 region), 3 (α1 and α2 region), 4 (α9 and α10 region), 5 (β1 and β2 region), 6 (The linker between α3 and β7), 7 (α11 and β7 region), and 1' (α4 and α6 region). The cumulative intercommunity betweenness is illustrated by the thickness of the lines connecting the communities.

Next, we performed a dynamic community analysis for the three FimX^EAL^ systems to gain deeper insights into the impact of ligand binding on the internal dynamics of the FimX^EAL^ protein. This analysis partitions the protein into distinct communities, represented by diverse colored circles in Figure 10, which are composed of interconnected nodes. The thickness of the edges connecting the communities illustrates the strength of the dynamic coupling between them.

As depicted in Figure 10, seven significant communities were identified in all three FimX^EAL^ systems, exhibiting a similar overall framework. However, closer examination reveals notable differences in the inter-community coupling patterns between the ligand-bound and apo systems. Compared to the FimX^EAL^-CDG and FimX^EAL^-MYR systems, the FimX^EAL^-apo system displayed an increased number of communities, suggesting a decrease in the inter-coupling within the FimX^EAL^ protein in the absence of ligand binding.

Interestingly, node 1, which is present as a single cohesive unit in the FimX^EAL^-CDG and FimX^EAL^-MYR systems, is fragmented into two smaller nodes (α2 and α4) in the FimX^EAL^-apo system. This fragmentation is particularly significant because α2 and α4 correspond to the binding regions for c-di-GMP and myricetin, respectively. The separation of these nodes in the apo system implies that the binding of c-di-GMP and myricetin plays a crucial role in bridging and enhancing the internal coupling within the FimX^EAL^ binding pocket. Therefore, the dynamic community analysis suggests that c-di-GMP and myricetin act as molecular bridges, strengthening the communications around the binding pocket and promoting a more cohesive and stable conformational state.

## 4. Discussion

*P. aeruginosa* has been identified as a constituent member of the ESKAPE pathogen consortium (*E. faecium*, *S. aureus*, *K. pneumoniae*, *A. baumannii*, *P. aeruginosa*, and *Enterobacter spp*.), with clinical infections caused by this organism demonstrating mortality rates ranging from 20% to 60% across diverse disease manifestations [43]. Biofilm formation has been recognized as a principal determinant underlying the escalation of antibiotic resistance and persistence phenotypes in *P. aeruginosa*. These structurally complex consortia, comprising surface-adhered microbial aggregates, are widely acknowledged as pivotal mediators of antimicrobial therapeutic failure. Functioning as a defensive matrix, biofilms enable bacterial populations to establish multilayered protection against diverse antimicrobial agents. Notably, epidemiological analyses have demonstrated that biofilm-associated infections account for over 80% of chronic bacterial pathogenesis cases [8,44]. Consequently, the escalating global healthcare challenge posed by multidrug-resistant (MDR) *P. aeruginosa* infections necessitates the development of innovative biofilm-targeted therapeutic interventions to counteract microbial adhesion capacity and overcome antimicrobial resistance.

Current anti-biofilm strategies can be categorized into three principal dimensions: (i) surface functionalization modifications to engineer anti-adhesive interfaces for preventing initial microbial attachment; (ii) pharmacological disruption of quorum sensing pathways to impede biofilm maturation; and (iii) mechanical biofilm eradication strategies employing physical/chemical energy modalities to degrade established matrices [44]. The developmental trajectory of microbial biofilms is mechanistically linked to bacterial motility phenotypes, as exemplified by the critical role of *P. aeruginosa* motile behaviors—encompassing swimming, swarming, and twitching motility—in mediating surface colonization, biofilm maturation, and infection initiation. These coordinated processes ultimately facilitate the emergence of multidrug resistance (MDR) through enhanced ecological adaptability and antimicrobial evasion mechanisms [45].

Notably, the regulation of motility-biofilm transition is closely associated with the second messenger cyclic di-guanosine monophosphate (c-di-GMP). The synthesis and degradation of c-di-GMP are controlled by diguanylate cyclases (DGCs) and phosphodiesterases (PDEs), respectively. Through this dynamic equilibrium, c-di-GMP orchestrates multiple critical physiological processes in bacteria, including biofilm formation, motility regulation, virulence factor expression, growth, and reproduction, as well as phenotypic switching [10]. Experimental studies have demonstrated that traditional Chinese medicine (TCM) components, such as triterpenoid saponins, coumarins, and raffinose, can function as c-di-GMP signaling inhibitors. These natural compounds effectively suppress biofilm formation and dispersal, offering a promising strategy for combating biofilm-associated infections [26]. Therefore, given the pivotal role of c-di-GMP signaling in biofilm formation, targeting c-di-GMP metabolism represents a promising strategy for developing novel anti-biofilm agents from traditional medicinal compounds. This study investigates the synergistic interactions between myricetin—a bioactive flavonoid derived from *Polygonum capitatum*—and multiple antibiotics (azithromycin/cefdinir/ciprofloxacin) through the lens of c-di-GMP signaling. Specifically, we elucidate how these combinations modulate *P. aeruginosa*’s resistance mechanisms by perturbing c-di-GMP homeostasis.

Myricetin exhibits broad-spectrum pharmacological activities, functioning as an effective biofilm inhibitor. Furthermore, it demonstrates synergistic antibacterial effects when combined with levofloxacin hydrochloride and sulfamethoxazole, highlighting its potential as a combinatorial therapeutic agent against biofilm-associated infections [46,47,48,49]. Data from FICI indices and time-kill curves demonstrate synergistic interactions between myricetin and antibiotics (azithromycin, cefdinir, and ciprofloxacin) against *P. aeruginosa* and its drug-resistant clinical strains, with MIC reductions by 2- to 32-fold. These findings warrant further investigations to determine whether c-di-GMP signaling serves as the target of myricetin’s dual-action mechanism combining anti-biofilm activity and resistance reversal in *P. aeruginosa*.

Bacterial motility plays a critical role in microcolony establishment and biofilm development in *P. aeruginosa* [50]. In this pathogen, c-di-GMP acts as a central regulator of motility, governing the transition between bacterial lifestyles through concentration-dependent mechanisms: elevated c-di-GMP levels drive biofilm formation and reinforce sessile colonization, whereas reduced concentrations promote a planktonic single-cell state and activate motility-associated behaviors [51]. Previous studies have demonstrated that cinnamaldehyde effectively suppresses motility and subsequently inhibits biofilm formation in *P. aeruginosa* by reducing c-di-GMP concentrations [50]. The findings of this experimental study revealed that co-treatment with sub-MIC concentrations of azithromycin, cefdinir, and ciprofloxacin in combination with myricetin resulted in a significant reduction of intracellular c-di-GMP levels in *P. aeruginosa*. Concurrently, this combinatorial treatment significantly inhibited bacterial swimming, swarming, and twitching motilities, suggesting that myricetin interferes with flagellar and type IV pili functionality, thereby impairing both biofilm formation and structural integrity.

To further elucidate the mechanism of action of myricetin, this study employed multiscale computational methods, including molecular docking, molecular dynamics simulations, and principal component analysis, to analyze the interactions between myricetin and the FimX target of c-di-GMP in *P. aeruginosa.* FimX is essential for normal twitching motility and biofilm formation in the opportunistic pathogen *P. aeruginosa* [51]. As a high-affinity receptor for c-di-GMP, FimX also regulates swarming motility [18]. Its degenerate EAL domain binds c-di-GMP and subsequently coordinates with the ATPase PilB to facilitate type IV pili assembly, thereby modulating bacterial motility [52]. The molecular docking results of this study suggest that myricetin can effectively bind to the FimX target and interfere with its regulation in bacterial motility and biofilm formation by altering the function of c-di-GMP. Principal component analysis and dynamic network analysis further confirmed the impact of myricetin on the FimX target, which may inhibit bacterial motility and biofilm formation by reducing the levels of c-di-GMP. Additionally, the binding free energy calculation results show that myricetin has a strong affinity for the FimX target, further supporting its potential as a regulatory factor for c-di-GMP.

## 5. Conclusions

The experimental results demonstrated that myricetin suppresses intracellular c-di-GMP biosynthesis in *P. aeruginosa*, effectively reducing antibiotic tolerance thresholds of both wild-type strains and clinically resistant isolates toward azithromycin, cefdinir, and ciprofloxacin. Synergistic interactions between myricetin and antibiotics were identified to enhance bactericidal efficacy and biofilm eradication potential. Furthermore, experimental validation through tri-modal motility assays (swimming, swarming, twitching) revealed pronounced inhibitory effects of myricetin on *P. aeruginosa* movement capacities. By integrating phenotypic screening with computational simulations, we characterize myricetin as a prototype adjuvant capable of recalibrating *P. aeruginosa* virulence in resistance-prone environments. These results propose c-di-GMP-targeted anti-biofilm strategies as an innovative solution to combat escalating antibiotic resistance, offering a dual-action therapeutic framework that synergizes biofilm disruption with resistance reversal mechanisms.

## Figures and Tables

**Table 1:** MIC values of myricetin and antibiotics against different strains (μg/mL).

Strains		Myricetin	Azithromycin	Ciprofloxacin	Cefdinir
***P. aeruginosa***	ATCC 9027	512	32	32	256
	PA01	512	128	128	1024
	PA02	512	256	256	>1024
	PA03	512	256	256	256
	PA04	1024	512	256	>1024
***E. coli***	CICC 10389	256	128	0.5	128
	EC01	1024	32	32	1024
	EC02	512	128	32	256
	EC03	1024	256	128	0.5
	EC04	1024	8	32	16
	EC05	1024	4	0.5	256
	EC06	1024	64	512	256
	EC07	256	8	0.5	256
***E. faecalis***	ATCC 19433	64	128	0.5	0.5
	EF01	1024	>1024	64	>1024
	EF02	1024	1024	256	128
	EF03	512	8	2	16
	EF04	1024	64	128	16
	EF05	1024	512	64	0.5

**Table 2:** Inhibition zone diameters for myricetin and different antibiotics alone and in combination against *P. aeruginosa* ATCC 9027.

Compounds	MYR	AZM	CIP	CFD	MYR + AZM	MYR + CIP	MYR + CFD
Inhibition Zone (mm)	-	-	26.0 ± 0.4	22.8 ± 0.6	13.7 ± 0.7 **	30.5 ± 0.9 **	24.9 ± 0.4 **

- No activity. ** The double (**) asterisks represented *p* <  0.01, compared to the corresponding antibiotic used alone.

**Table 3:** Fractional inhibitory concentration index (FICI) results for the combination of myricetin and antibiotics against both standard and clinically resistant strains of *P. aeruginosa*.

Strains	MIC (μg/L) in Combination		Potentiation ^a^	FICI	Inf	MIC (μg/L) in Combination		Potentiation	FICI	Inf	MIC (μg/L) in Combination		Potentiation	FICI	Inf
	MYR	AZM				MYR	CIP				MYR	CFD			
ATCC 9027	128	4	8-fold	0.375	SN	256	16	2-fold	1	AD	256	64	4-fold	0.75	PS
PA01	32	8	16-fold	0.125	SN	16	32	4-fold	0.28125	SN	64	32	32-fold	0.15625	SN
PA02	256	32	8-fold	0.625	PS	256	256	1-fold	1.5	AN	256	64	16-fold	0.5625	PS
PA03	256	16	16-fold	0.5625	PS	512	128	2-fold	1.5	AN	256	64	4-fold	0.75	PS
PA04	256	16	32-fold	0.28125	SN	256	16	16-fold	0.3125	SN	256	64	4-fold	0.5	SN

^a^ Multiples of MIC reduction compared with the antibiotic alone. Inf-inference; SN-synergy; PS-partial synergy; AD-additive; AN-antagonism; MYR-myricetin; AZM-azithromycin; CIP-ciprofloxacin; CFD-cefdinir.

**Table 4:** Components of the binding free energy between FimX^EAL^ and two ligands (c-di-GMP and myricetin) averaged in the last 100 ns of the three replicates (kcal/mol).

Contribution	FimX^EAL^-CDG	FimX^EAL^-MYR
Δ*E*_vdw_	−51.12 ± 3.08	−28.13 ± 1.44
Δ*E*_ele_	53.34 ± 7.38	−59.69 ± 0.72
Δ*G*_pol,sol_	−35.46 ± 5.70	64.19 ± 0.58
Δ*G*_npol,sol_	−6.17 ± 0.38	−4.62 ± 0.15
Δ*E*_MM_	2.23 ± 8.91	−87.81 ± 1.87
Δ*G*_sol_	−41.63 ± 5.57	59.57 ± 0.46
Δ*G*_MM/GBSA_	−39.40 ± 3.36	−28.24 ± 1.61

## Data Availability

The original contributions presented in this study are included in the article and Supplementary Material. Further inquiries can be directed to the corresponding authors.

## Supplementary Materials

The following supporting information can be downloaded at: https://www.mdpi.com/article/10.3390/biology14070859/s1, Figure S1: Inhibition zone diameters for myricetin and different antibiotics alone and in combination against *P. aeruginosa* ATCC 9027. Figure S2: Molecular docking analysis of myricetin with c-di-GMP effector proteins from *P. aeruginosa.* (A) FimX-myricetin complex (docking score: −7.315 kcal/mol), (B) BrlR-myricetin complex (docking score: −4.472 kcal/mol), and (C) FleQ-myricetin complex (docking score: −5.078 kcal/mol). Protein structures are shown in cartoon representation with FimX in green, BrlR in cyan, and FleQ in light purple. Myricetin (MYR) is displayed as a stick model in red. Figure S3: The RMSDs of all simulated systems during three independent 500-ns MD simulations: (A) FimX^EAL^-apo system; (B) FimX^EAL^-CDG system; (C) FimX^EAL^-MYR system. References [18,53,54,55,56,57,58,59,60,61,62,63,64,65,66] are cited in the Supplementary Materials.
