# Correction: Zeng et al. Myricetin Potentiates Antibiotics Against Resistant *Pseudomonas aeruginosa* by Disrupting Biofilm Formation and Inhibiting Motility Through FimX-Mediated c-di-GMP Signaling Interference. *Biology* 2025, *14*, 859

**DOI:** 10.3390/biology14121660

**Published:** 2025-11-24

**Authors:** Derong Zeng, Fangfang Jiao, Yuqi Yang, Shuai Dou, Jiahua Yu, Xiang Yu, Yongqiang Zhou, Juan Xue, Xue Li, Hongliang Duan, Yan Zhang, Jingjing Guo, Wude Yang

**Affiliations:** 1 College of Pharmacy, Guizhou University of Traditional Chinese Medicine, Guiyang 550025, China; 2 Centre in Artificial Intelligence Driven Drug Discovery, Faculty of Applied Sciences, Macao Polytechnic University, Macao, China; 3 School of Basic Medicine, Guizhou University of Traditional Chinese Medicine, Guiyang 550025, China; 4 The Second Affiliated Hospital of Guizhou University of Traditional Chinese Medicine, Guiyang 550025, China

**Error in Figure**

In the original publication [1], there was a mistake in Figure 6 as published. The image intended for Figure 6A (ATCC group, twitching row, and CIP + MYR column) was inadvertently duplicated in Figure 6C (PA01 group). The corrected Figure 6 appears below. The authors state that the scientific conclusions are unaffected. This correction was approved by the Academic Editor. The original publication has also been updated.

## Figures and Tables

**Figure 6:**
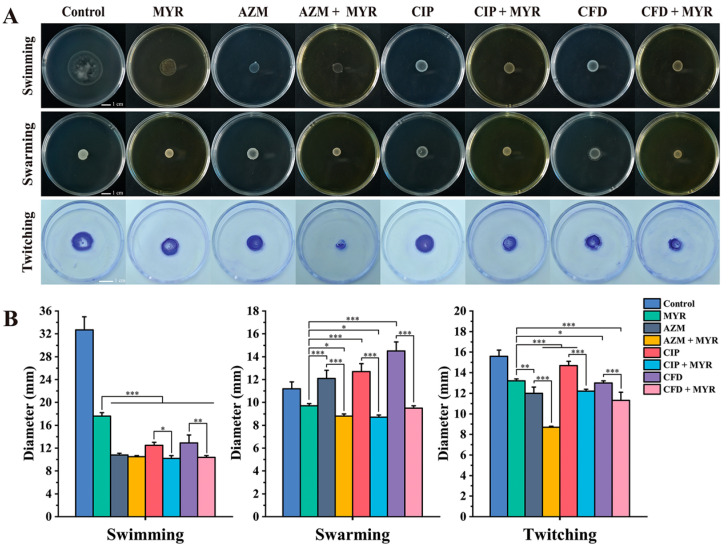

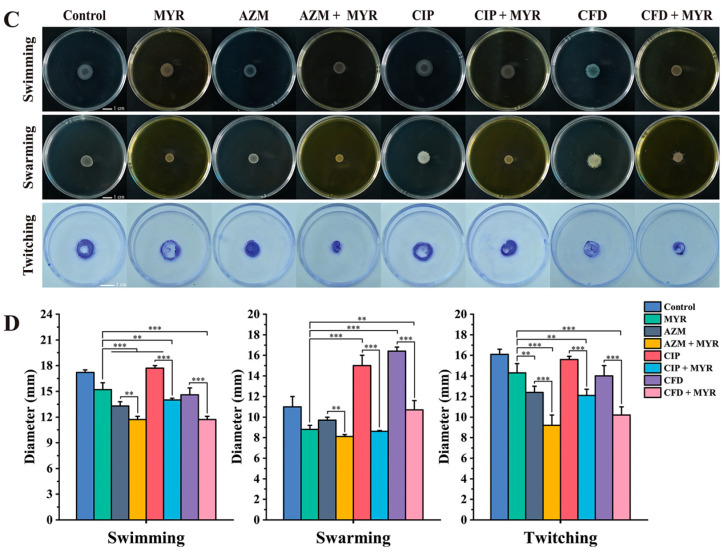
Effects of myricetin at 1/8 MIC concentration and antibiotics at 1/4 MIC concentration on the motility of *P. aeruginosa* ATCC 9027 and PA01. Swimming, swarming, and twitching motility of (**A**) ATCC 9027 group and (**C**) PA01 group. Statistics of swimming, swarming, and twitching motility diameters of (**B**) ATCC 9027 group and (**D**) PA01 group. Data are expressed as mean ± SD of at least three independent experiments (*, *p* < 0.05; **, *p* < 0.01; ***, *p* < 0.001).

## References

[1] Zeng D., Jiao F., Yang Y., Dou S., Yu J., Yu X., Zhou Y., Xue J., Li X., Duan H. (2025). Myricetin Potentiates Antibiotics Against Resistant *Pseudomonas aeruginosa* by Disrupting Biofilm Formation and Inhibiting Motility Through FimX-Mediated c-di-GMP Signaling Interference. Biology.

