# Supplementary material for: Myricetin Potentiates Antibiotics Against Resistant Pseudomonas aeruginosa by Disrupting Biofilm Formation and Inhibiting Motility Through FimX-Mediated c-di-GMP Signaling Interference
Source: Biology (Basel). 2025 Jul 15;14(7):859. doi: 10.3390/biology14070859 (PMC12292220; doi:10.3390/biology14070859)
Supplement: Supplementary file 1 [file biology-14-00859-s001.zip › biology-3706036-File S1.docx]

Supplementary Materials

Myricetin Potentiates Antibiotics Against Resistant
*Pseudomonas aeruginosa* by Disrupting Biofilm Formation and Inhibiting Motility Through FimX-Mediated c-di-GMP
Signaling Interference

Derong Zeng ^1,†^, Fangfang Jiao ^2,†^, Yuqi Yang ^3^, Shuai Dou ^1^, Jiahua Yu ^1^, Xiang Yu ^1^, Yongqiang Zhou ^1^, Juan Xue ^1^, Xue Li ^4^, Hongliang Duan ^2^, Yan Zhang ^1,^*, Jingjing Guo ^2,^* and Wude Yang ^1^

^1^ College of Pharmacy, Guizhou University of Traditional Chinese Medicine, Guiyang 550025, China;
zengderogs@foxmail.com (D.Z.); doushuaigzy@163.com (S.D.); yujiahua0410@163.com (J.Y.); yuxiangjx@126.com (X.Y.); zhouxiaoqiang1988@126.com (Y.Z.); xuejuan062@gzy.edu.cn (J.X.); yangwude476@gzy.edu.cn (W.Y.)

^2^ Centre in Artificial Intelligence Driven Drug Discovery, Faculty of Applied Sciences, Macao Polytechnic University, Macao, China; p2212238@mpu.edu.mo (F.J.); hduan@mpu.edu.mo (H.D.)

^3^ School of Basic Medicine, Guizhou University of Traditional Chinese Medicine, Guiyang 550025, China; yangyuqi061@gzy.edu.cn

^4^ The Second Affiliated Hospital of Guizhou University of Traditional Chinese Medicine,
Guiyang 550025, China; lixue199597@163.com

***** Correspondence: zhangyan0003@gzy.edu.cn (Y.Z.); jguo@mpu.edu.mo (J.G.)

^†^ These authors contributed equally to this work.

S1. Computational approaches

S1.1. System preparation

In this study, three distinct model systems were constructed based on the crystal structure of the FimX EAL domain (FimX^EAL^) in complex with the c-di-GMP molecule (PDB ID: 3HV8 [1]), hereafter referred to as FimX^EAL^-CDG. Initially, the missing residues (521-525) in the FimX^EAL^-CDG system were modeled using the Prime module in the Schrödinger [2] to ensure the structural integrity of the protein. Subsequently, the c-di-GMP molecule was removed from the FimX^EAL^-CDG complex to generate the apo state of FimX^EAL^, denoted as FimX^EAL^-apo. In addition, the myricetin molecule was introduced into the c-di-GMP binding site of FimX^EAL^ using the Glide module in Schrödinger[2], resulting in the FimX^EAL^-MYR system (detailed docking procedures are described in Section 1.3). In summary, three model systems were constructed (Figure 1): FimX^EAL^-apo, FimX^EAL^-CDG, and FimX^EAL^-MYR, which serve as the foundation for further molecular dynamics (MD) simulation.

S1.2. Molecular dynamics simulations

MD simulations were performed on three model systems, namely FimX^EAL^-apo, FimX^EAL^-CDG, and FimX^EAL^-MYR, using the AMBER 20 [3] package. The force field parameters and partial charges for c-di-GMP and myricetin were derived using the Antechamber module in AMBER 20, with the resp charge fitting method based on electrostatic potentials calculated using Gaussian 09. The ff14SB[4] and GAFF [5] force fields were used to describe the protein and small molecules, respectively. Each system was neutralized by adding an appropriate number of counterions (Cl^–^ or Na^+^) and then solvated with TIP3P [6] water molecules in a periodic orthogonal box, maintaining a minimum distance of 10 Å between the solute atoms and the box boundaries. A cutoff distance of 10 Å was set for non-bonded interactions [7], and long-range electrostatic interactions were handled using the particle mesh Ewald method. The Shake [8] algorithm was employed to constrain bonds involving hydrogen atoms.

The MD simulations were conducted in four stages: energy minimization, heating, equilibration, and production. During energy minimization, the systems were subjected to 27,000 steps of steepest descent and conjugate gradient minimization, with restraints on heavy atoms, protein backbone, and Cα atoms gradually reduced from 5.0 to 0 kcal/mol/Å^2^. The minimized systems were then heated from 0 K to 310 K over 200 ps, with a harmonic restraint of 5.0 kcal/mol/Å^2^ applied to all heavy atoms. Next, a 10-ns equilibration was performed in the NVT and NPT ensembles, with restraint forces gradually decreasing from 1.0 to 0.1 kcal/mol/Å^2^. Finally, three independent 500 ns production runs were carried out for each system, with randomized initial velocities assigned to the atoms in each replicate.

S1.3. Molecular docking

The crystal structure of three c-di-GMP effector proteins—FimX^EAL^(PDB ID: 3HV8 [1]), FleQ (PDB ID: 5EXX [9]), and BrlR (PDB ID: 5XBT [10])—in complex with c-di-GMP were selected as docking receptors. To prepare the protein receptor for docking, the Protein Preparation Wizard in Schrödinger was employed, which included adding hydrogens, optimizing hydrogen bonds, and minimizing the structure using the OPLS_2005 force field [2]. The myricetin molecule, serving as the docking ligand, was prepared using the LigPrep module in Schrödinger [2], which generated possible ionization states and tautomers, and minimized the structure. To define the docking search space, a grid box was centered on the c-di-GMP molecule in the FimX^EAL^ binding site, with dimensions set to accommodate the size of the myricetin molecule. Molecular docking was then performed using the Glide program in Schrödinger [2], with the extra precision [11] (XP) scoring function to prioritize the most energetically favorable and structurally complementary binding poses. The top-ranking docking pose of myricetin was selected for further MD simulations.

S1.4. Principal component analysis

Principal component analysis (PCA) was performed on the last 100 ns of the MD trajectories for each FimX^EAL^ system using the CPPTRAJ [12] module of AmberTools. In this study, the covariance matrix was calculated using the Cartesian coordinates of the Cα atoms of FimX^EAL^ after superimposing the trajectories onto their average structure to remove translational and rotational motions. The covariance matrix was then diagonalized to obtain the eigenvectors and eigenvalues, which represent the principal components (PCs) and their corresponding variances, respectively. The two most significant principal components (PC1 and PC2), which capture the largest proportion of the overall motion, were selected to define a reduced dimensional space. Each conformation in the trajectories was then projected onto this PC1-PC2 subspace to visualize the conformational distribution of FimX^EAL^. The relative populations of conformations in the PC1-PC2 subspace were estimated using the Boltzmann [13] distribution, which relates the probability of a conformation to its energy.

S1.5. Binding free energy calculation

To gain further insights into the binding affinity of FimX^EAL^ with c-di-GMP and myricetin, the Molecular Mechanics/Generalized Born Surface Area (MM/GBSA) method was employed to calculate the binding free energies. In this study, snapshots were extracted from the last 100-ns MD trajectories at regular intervals, excluding water molecules and counterions. The binding free energy (Δ*G*_bind_) for each snapshot was calculated as the difference between the free energy of the complex (Δ*G*_complex_) and the sum of the free energies of the receptor (Δ*G*_receptor_) and the ligand (Δ*G*_ligand_):

$$\text{∆}\text{G}_{\text{bind}}\text{ }\text{=}\text{ ∆}\text{G}_{\text{complex}}-\text{∆}\text{G}_{\text{receptor}}-\text{∆}\text{G}_{\text{ligand}}$$

The free energy terms were computed using a combination of molecular mechanics energy terms (including bond, angle, dihedral, van der Waals, and electrostatic energies) and polar and nonpolar solvation free energies estimated using the Generalized Born (GB) model and solvent-accessible surface area (SASA) calculations, respectively. Entropic contributions to the binding free energy were not considered in this study, as the focus was on the relative binding affinities rather than absolute Gibbs free energies. The final reported Δ*G*_bind_ values represent the mean and standard error of the mean (SEM) calculated over the three independent simulations for each system.

S1.6. Protein dynamical network analysis

To investigate the effect of ligand binding on the dynamic properties of FimX^EAL^, community network analysis was performed using the NetworkView [14] plugin in Visual Molecular Dynamics [15] (VMD). This approach converts the protein structure into a network representation, where nodes correspond to the Cα atoms of residues and edges represent the strength of residue-residue interactions. In this study, the community network analysis was performed on the last 100-ns MD trajectory for the three FimX^EAL^ systems, considering three replicates per system to ensure statistical robustness.

The first step in the community network analysis involves the construction of the residue interaction network. An edge is established between two nodes (residues) if any heavy atom of one residue is within a cutoff distance of 4.5 Å from any heavy atom of the other residue for at least 75% of the simulation time. This criterion ensures that only persistent interactions are considered in the network. The edge weights are determined based on the correlation between the dynamic fluctuations of the connected residues, capturing the strength of their coupling. Next, the Girvan-Newman algorithm is employed to partition the network into communities, which are groups of highly interconnected nodes. The algorithm iteratively removes edges with high betweenness centrality, which measures the number of shortest paths passing through an edge, until the network is divided into non-overlapping communities. A smaller number of communities indicates stronger coupling and more efficient communication within the protein. The final community network is obtained by integrating the residue-residue interactions and correlations over the entire MD trajectory.


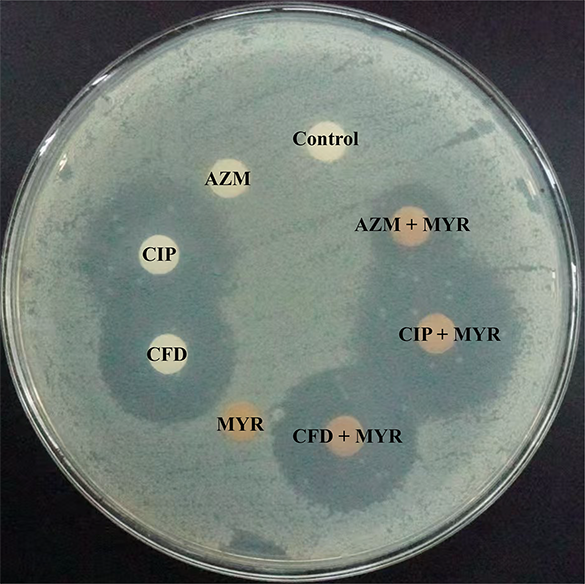


**Figure S1.** Inhibition zone diameters for myricetin and different antibiotics alone and in combination against *P. aeruginosa* ATCC 9027.


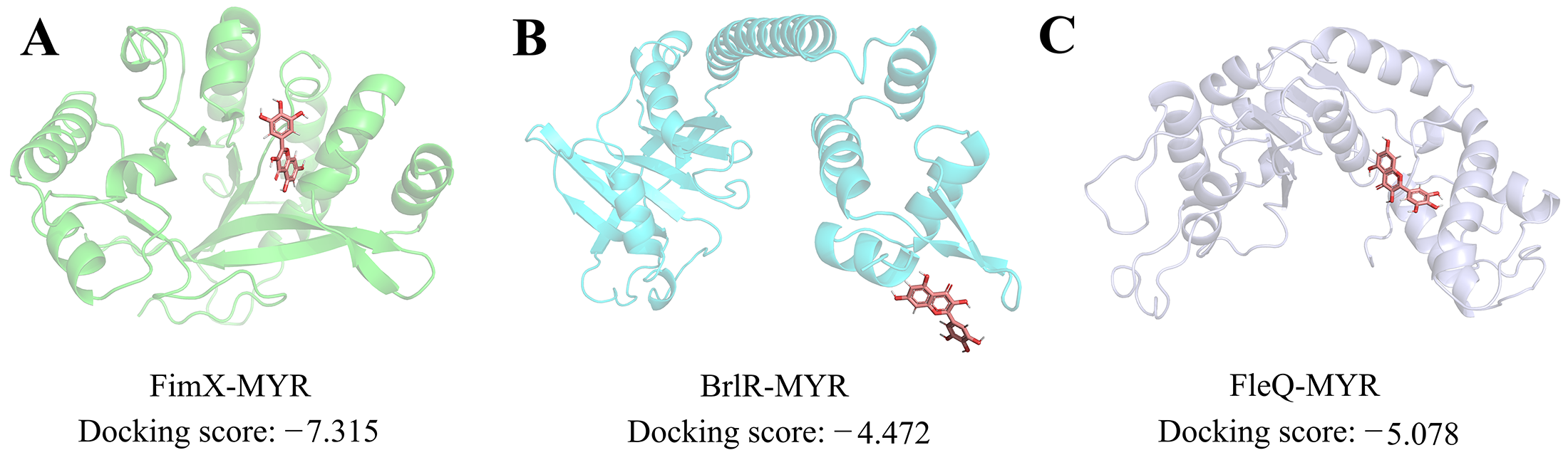


**Figure S2.** Molecular docking analysis of myricetin with c-di-GMP effector proteins from *P. aeruginosa.* Three-dimensional representations of the docking complexes between myricetin and three c-di-GMP effector proteins: (A) FimX-myricetin complex (docking score: −7.315 kcal/mol), (B) BrlR-myricetin complex (docking score: −4.472 kcal/mol), and (C) FleQ-myricetin complex (docking score: −5.078 kcal/mol). Protein structures are shown in cartoon representation with FimX in green, BrlR in cyan, and FleQ in light purple. Myricetin (MYR) is displayed as a stick model in red.


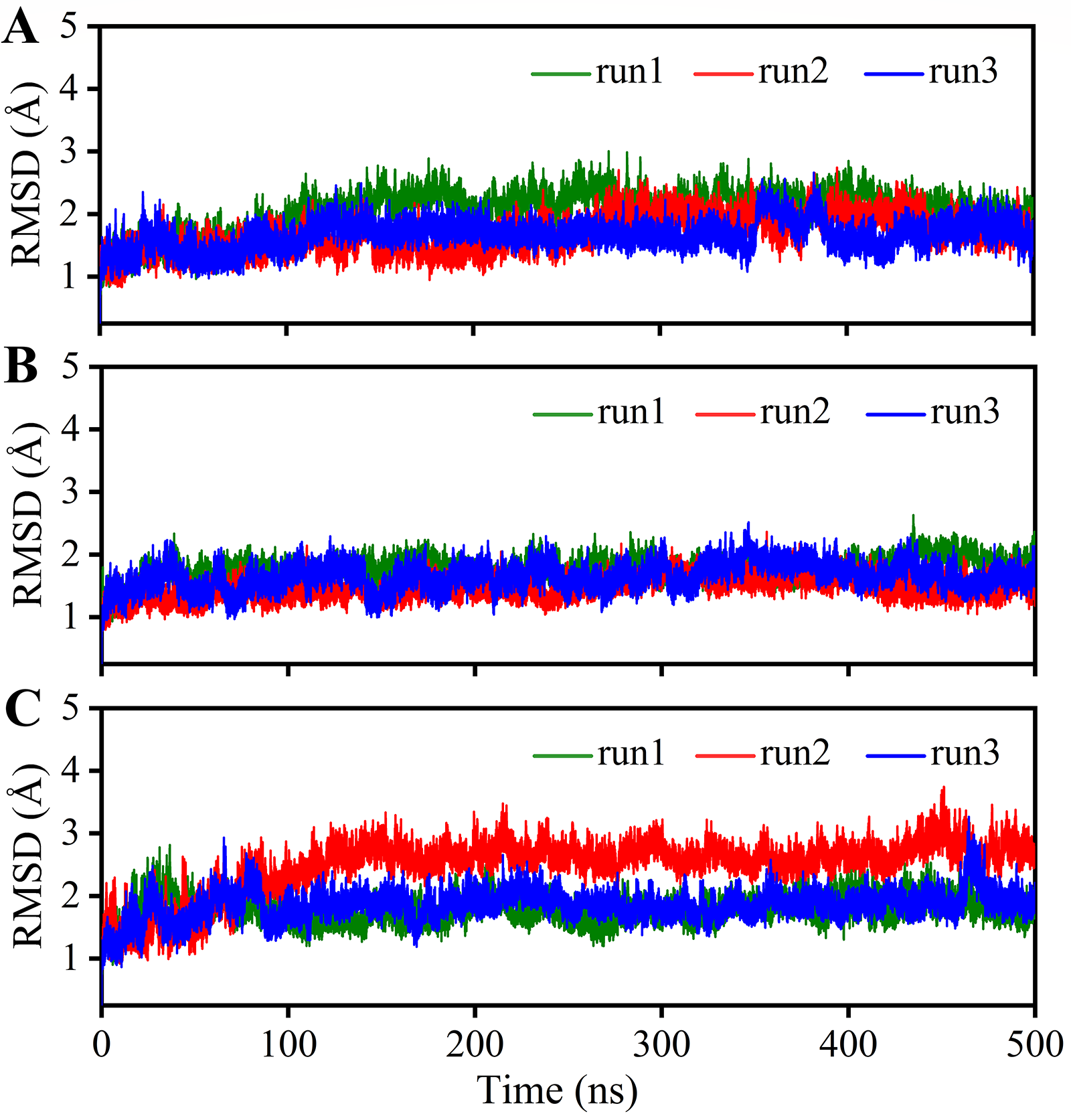


**Figure S3.** The RMSDs of all simulated systems during three independent 500-ns MD simulations: (A) FimX^EAL^-apo system; (B) FimX^EAL^-CDG system; (C) FimX^EAL^-MYR system.

References

1. Navarro, M.V.A.S.; De, N.; Bae, N.; Wang, Q.; Sondermann, H. Structural Analysis of the GGDEF-EAL Domain-Containing c-di-GMP Receptor FimX. *Structure* **2009**, *17*, 1104–1116. https://doi.org/10.1016/j.str.2009.06.010.
2. Sastry, G.M.; Adzhigirey, M.; Day, T.; Annabhimoju, R.; Sherman, W. Protein and ligand preparation: Parameters, protocols, and influence on virtual screening enrichments. *J. Comput. Aided Mol. Des.* **2013**, *27*, 221–234. https://doi.org/10.1007/s10822-013-9644-8.
3. Case, D.A.; Cheatham, T.E.; Darden, T.; Gohlke, H.; Luo, R.; Merz, K.M.; Onufriev, A.; Simmerling, C.; Wang, B.; Woods, R.J. The Amber biomolecular simulation programs. *J. Comput. Chem.* **2005**, *26*, 1668–1688. https://doi.org/10.1002/jcc.20290.
4. Maier, J.A.; Martinez, C.; Kasavajhala, K.; Wickstrom, L.; Hauser, K.E.; Simmerling, C. ff14SB: Improving the accuracy of protein side chain and backbone parameters from ff99SB. *J. Chem. Theory Comput*. **2015**, *11*, 3696–3713. https://doi.org/10.1021/acs.jctc.5b00255.
5. Wang, J.; Wolf, R.M.; Caldwell, J.W.; Kollman, P.A.; Case, D.A. Development and testing of a general amber force field. *J. Comput. Chem.* **2004**, *25*, 1157–1174. https://doi.org/10.1002/jcc.20035.
6. Jorgensen, W.L.; Chandrasekhar, J.; Madura, J.D.; Impey, R.W.; Klein, M.L. Comparison of simple potential functions for simulating liquid water. *J. Chem. Phys.* **1983**, *79*, 926–935. https://doi.org/10.1063/1.445869.
7. Essmann, U.; Perera, L.; Berkowitz, M.L.; Darden, T.; Lee, H.; Pedersen, L.G. A smooth particle mesh Ewald method. *J. Chem. Phys.* **1995**, *103*, 8577–8593. https://doi.org/10.1063/1.470117.
8. Ryckaert, J.-P.; Ciccotti, G.; Berendsen, H.J.C. Numerical integration of the cartesian equations of motion of a system with constraints: Molecular dynamics of n-alkanes. *J. Comput. Phys.* **1977**, *23*, 327–341. https://doi.org/10.1016/0021-9991(77)90098-5.
9. Matsuyama, B.Y.; Krasteva, P.V.; Baraquet, C.; Harwood, C.S.; Sondermann, H.; Navarro, M.V.A.S. Mechanistic insights into c-di-GMP–dependent control of the biofilm regulator FleQ from*Pseudomonas aeruginosa*. *Proc. Natl. Acad. Sci. USA* **2015**, *113*, E209–E218. https://doi.org/10.1073/pnas.1523148113.
10. Wang, F.; He, Q.; Yin, J.; Xu, S.; Hu, W.; Gu, L. BrlR from Pseudomonas aeruginosa is a receptor for both cyclic di-GMP and pyocyanin. *Nat. Commun.* **2018**, *9*, 2563. https://doi.org/10.1038/s41467-018-05004-y.
11. Friesner, R.A.; Murphy, R.B.; Repasky, M.P.; Frye, L.L.; Greenwood, J.R.; Halgren, T.A.; Sanschagrin, P.C.; Mainz, D.T. Extra Precision Glide: Docking and Scoring Incorporating a Model of Hydrophobic Enclosure for Protein−Ligand Complexes. *J. Med. Chem.* **2006**, *49*, 6177–6196. https://doi.org/10.1021/jm051256o.
12. Roe, D.R.; Cheatham, T.E. PTRAJ and CPPTRAJ: Software for Processing and Analysis of Molecular Dynamics Trajectory Data. *J. Chem. Theory Comput.* **2013**, *9*, 3084–3095. https://doi.org/10.1021/ct400341p.
13. He, X.; Luo, L.-S. Theory of the lattice Boltzmann method: From the Boltzmann equation to the lattice Boltzmann equation. *Phys. Rev. E* **1997**, *56*, 6811–6817. https://doi.org/10.1103/physreve.56.6811.
14. Eargle, J.; Luthey-Schulten, Z. *NetworkView*: 3D display and analysis of protein·RNA interaction networks. *Bioinformatics* **2012**, *28*, 3000–3001. https://doi.org/10.1093/bioinformatics/bts546.
15. Humphrey, W.; Dalke, A.; Schulten, K. VMD: Visual Molecular Dynamics. *J. Mol. Graph.* **1996**, *14*, 33–38.

**Disclaimer/Publisher’s Note:** The statements, opinions and data contained in all publications are solely those of the individual author(s) and contributor(s) and not of MDPI and/or the editor(s). MDPI and/or the editor(s) disclaim responsibility for any injury to people or property resulting from any ideas, methods, instructions or products referred to in the content.
